# Editorial: Management of right ventricular failure: pathophysiology, medical treatment and use of ventricular assist devices

**DOI:** 10.3389/fcvm.2023.1297652

**Published:** 2023-11-09

**Authors:** Toni Soriano-Colomé, José Antonio Barrabés, Sofie Gevaert, Antonia Sambola

**Affiliations:** ^1^ Department of Cardiology, University Hospital Vall d'Hebron, University Autonomous of Barcelona, Barcelona, Spain; ^2^ Department of Cardiology, Research Institut Vall d’Hebron, Barcelona, Spain; ^3^ Department of Cardiology, CIBERCV, Barcelona, Spain; ^4^ Department of Cardiology, Ghent University Hospital, Ghent, Belgium

**Keywords:** LVAD (left ventricular assist device), right ventricular (RV) dysfunction, right ventricular (RV) failure, advanced heart failure (AHF), management - intensive care

**Editorial on the Research Topic** Management of right ventricular failure: pathophysiology, medical treatment and use of ventricular assist devices

The right ventricle (RV) has a crucial role in the evolution and prognosis of multiple cardiovascular conditions. The RV is anatomically and functionally different from the left ventricle (LV). RV adaptation to hemodynamic changes is determined by hemodynamic overload as well as by its intrinsic contractile function. The close relationship between both the RV and LV (ventricular interdependence) and the pulmonary circulation further modulates RV behavior (1). This research topic includes four articles focused on RV failure in different scenarios (Hao et al., Zhang et al., Bravo et al.) (2).

Hao et al. present a case report of postoperative RV dysfunction in a woman with adult congenital heart disease (coarctation of the aorta and bicuspid aortic valve) who presented with a type-A aortic dissection and an ascending aortic aneurysm (diameter: 71 mm) at 20 weeks of gestation. The patient underwent Bentall's surgery, but unfortunately the fetus died in the postoperative period. After C-section, the woman developed cardiogenic shock (CS). The differential diagnosis included: amniotic liquid embolism, pulmonary embolism, and myocardial infarction (MI). The final diagnosis was MI with right coronary artery occlusion. Echocardiography showed mild depression of LV function and severe RV dysfunction that was successfully treated with vasoactive drugs. This case shows the importance of diagnosis of RV dysfunction in different aetiologies of CS. On the other hand, it is worth highlighting that the woman was in WHO grade IV classification, in which case surgical is indicated before pregnancy.

Zhang et al. explore the value of RV strain as a tool for estimating RV function (1). The aim of this study was to relate RV-free wall longitudinal strain before aortic valvular substitution with low cardiac output (CO) after surgery, as well as the risk of readmission at two years in patients with low CO. This is a single-center, retrospective, observational study including 146 patients and 12% of these met the criteria for low CO. A cut-off point of −18.3% for RV-free wall strain was a predictor of low postoperative CO with an AUC of 0.879. Although the study has some limitations, this cut-off could be useful to identify patients at high risk of postoperative RV failure. In contrast, neither TAPSE nor other classic measures of RV systolic function were independent predictors of poor CO, suggesting that the strain is a more sensitive in parameter to detect patients at risk.

Early RV failure is a devastating complication occurring after LVAD implantation, and it is quite common, reaching 20%–40% of cases in various series and requiring mechanical RV support in up to 5% of cases. Early RV failure is associated with higher mortality, longer hospital stays, and increased costs. Late post-LVAD RV failure in outpatients results in higher readmission rates, mortality, and pump-related thrombotic events.

The new MCS-ARC definitions of 2020 classify post-LVAD RV failure as early (<30 days) or late (>30 days), and late RV is defined as the presence of signs and symptoms of RV failure in combination with an increase of diuretics and/or a need for inotropes for at least 72 h (2).

Two manuscripts focus on extensive reviews of the failure of RV post-LVAD (Bravo et al., Rodenas-Alesina et al.). The article by Rodenas-Alesina et al. is an excellently structured guidance focused on the evaluation of RV failure post-LVAD implantation. Predictive preoperative assessment based on clinical features and a combination of echocardiographic and hemodynamic RV metrics is essential to detect patients with high risk of RV failure. Other intraoperative measures to avoid RV failure post-LVAD implantation are reduction of bleeding events, prevention of coagulopathy, avoidance of pericardiotomy and cardiopulmonary bypass, repair of valvular insufficiencies, and treatment of coronary artery disease if present.

Bravo et al. and Rodenas-Alesina et al. both cover the early management of post-LVAD RV failure, based on observational studies. Management consists of early extubation, optimization of blood volume, avoiding high CVP and being aggressive with diuretic treatment and renal replacement techniques if necessary, use of pulmonary vasodilators, inotropic treatment: dobutamine and milrinone, the latter may be preferred for its pulmonary vasodilator effect and control of sinus rhythm and heart rate (HR) trying to maintain high HR to improve RV CO, through epicardial pacemaker or endocardial stimulation if the patient has a device. The last resort when other therapies fail is mechanical support (MS) of the RV. The specific device should be selected, based on the level of support needed and the center's experience.

Late RV failure is relatively common, with moderate-severe episodes occurring in 20% of patients at one month and 3%–5% at three months (1). The management is individualized, although it normally involves reduction of the LVAD speed. Yet, a ramp test guided by image or right catheterization could be useful to find the most appropriate speed. Maintenance of treatment for LV dysfunction is recommended, as well as accurate volume management including RRT techniques and peritoneal dialysis. Intermittent ambulatory infusions of inotropes, especially levosimendan, represent an alternative approach.

We would like to finish this Research Topic with a call for action to perform more studies on different aspects of RV failure post-LVAD implantation. Significant gaps remain in our understanding and optimal treatment of this pathology.
